# Prognostic factors in Spanish COVID-19 patients: A case series from Barcelona

**DOI:** 10.1371/journal.pone.0237960

**Published:** 2020-08-21

**Authors:** Antoni Sisó-Almirall, Belchin Kostov, Minerva Mas-Heredia, Sergi Vilanova-Rotllan, Ethel Sequeira-Aymar, Mireia Sans-Corrales, Elisenda Sant-Arderiu, Laia Cayuelas-Redondo, Angela Martínez-Pérez, Noemí García-Plana, August Anguita-Guimet, Jaume Benavent-Àreu

**Affiliations:** 1 Consorci d’Atenció Primària de Salut Barcelona Esquerra (CAPSBE), Barcelona, Spain; 2 Primary Healthcare Transversal Research Group, Institut d’Investigacions Biomèdiques August Pi i Sunyer (IDIBAPS), Barcelona, Spain; 3 Primary Care Centre Les Corts, Barcelona, Spain; 4 Primary Care Centre Casanova, Barcelona, Spain; 5 Primary Care Centre Comte Borrell, Barcelona, Spain; Osakidetza Basque Health Service, SPAIN

## Abstract

**Background:**

In addition to the lack of COVID-19 diagnostic tests for the whole Spanish population, the current strategy is to identify the disease early to limit contagion in the community.

**Aim:**

To determine clinical factors of a poor prognosis in patients with COVID-19 infection.

**Design and setting:**

Descriptive, observational, retrospective study in three primary healthcare centres with an assigned population of 100,000.

**Method:**

Examination of the medical records of patients with COVID-19 infections confirmed by polymerase chain reaction. Logistic multivariate regression models adjusted for age and sex were constructed to analyse independent predictive factors associated with death, ICU admission and hospitalization.

**Results:**

We included 322 patients (mean age 56.7 years, 50% female, 115 (35.7%) aged ≥ 65 years): 123 (38.2) were health workers (doctors, nurses, auxiliaries). Predictors of ICU admission or death were greater age (OR = 1.05; 95%CI = 1.03 to 1.07), male sex (OR = 2.94; 95%CI = 1.55 to 5.82), autoimmune disease (OR = 2.82; 95%CI = 1.00 to 7.84), bilateral pulmonary infiltrates (OR = 2.86; 95%CI = 1.41 to 6.13), elevated lactate-dehydrogenase (OR = 2.85; 95%CI = 1.28 to 6.90), elevated D-dimer (OR = 2.85; 95%CI = 1.22 to 6.98) and elevated C-reactive protein (OR = 2.38; 95%CI = 1.22 to 4.68). Myalgia or arthralgia (OR = 0.31; 95%CI = 0.12 to 0.70) was protective factor against ICU admission and death. Predictors of hospitalization were chills (OR = 5.66; 95%CI = 1.68 to 23.49), fever (OR = 3.33; 95%CI = 1.89 to 5.96), dyspnoea (OR = 2.92; 95%CI = 1.62 to 5.42), depression (OR = 6.06; 95%CI = 1.54 to 40.42), lymphopenia (OR = 3.48; 95%CI = 1.67 to 7.40) and elevated C-reactive protein (OR = 3.27; 95%CI = 1.59 to 7.18). Anosmia (OR = 0.42; 95%CI = 0.19 to 0.90) was the only significant protective factor for hospitalization after adjusting for age and sex.

**Conclusion:**

Determining the clinical, biological and radiological characteristics of patients with suspected COVID-19 infection will be key to early treatment and isolation and the tracing of contacts.

## Introduction

On 31 December 2019, the health authorities of Wuhan city (Hubei Province, China) reported a cluster of 27 cases of pneumonia of unknown aetiology with onset of symptoms on 8 December, including 7 severe cases, with a common exposure identified in a city market [1], which was closed on January 1, 2020. On 7 January 2020, the Chinese authorities identified a new *Coronaviridae* family virus, initially named coronavirus 2019-nCoV and later coronavirus SARS-CoV-2 as the causal agent [2]. The genetic sequence was shared by the Chinese authorities on 12 January 2020. On January 19, the first case was detected in the USA, in Washington state [3]. On 30 January 2020, the World Health Organization declared the SARS-CoV-2 outbreak in China a public health emergency of international concern [4]. Subsequently, the outbreak has spread outside China, with Europe especially affected [5].

The first positive case diagnosed in Spain was confirmed on January 31, 2020 on the island of La Gomera, while the first death occurred on February 13 in Valencia city (the date was confirmed twenty days later). The first confirmed case in Barcelona was on 24 February, and from then until 29 June 2020, there have been 248,970 confirmed cases in Spain [6].

The most common signs of infection are respiratory symptoms: fever, cough and shortness of breath. In more severe cases, the infection may cause pneumonia, severe acute respiratory syndrome, renal failure and death [7]. Transmission appears to be mainly person-to-person via the airway through respiratory droplets measuring > 5 microns when the patient has respiratory symptoms (cough and sneezing) and contact with fomites [8]. Most estimates of the incubation period of COVID-19 range from 1 to 14 days, with most around five days. Evidence on the transmission of the virus before symptom onset is unclear. There is currently no specific treatment for COVID-19 infections. To date, the most important scientific efforts have focused on three areas: strategies to contain the spread of the disease, the initiation of clinical trials with antivirals and multiple therapies, and the design of a new vaccine, which is still unclear. These strategies include some of a community nature, where primary healthcare plays a central role in disease prevention and control [9]. Few studies have described the clinical characteristics of the disease, fewer the predictive factors, and virtually none have described the Mediterranean population compared with the rest of the world. Therefore, this study aimed to describe the clinical, biological and radiological manifestations, the evolution, treatments and mortality rate of patients with COVID-19 infection in the population of Barcelona city and determine the most important predictors of a poor prognosis.

## Materials and methods

A multicentre, observational descriptive study was carried out in three urban primary healthcare centres serving an assigned population of 100,000, with one reference hospital. The study included all consecutive adult patients with COVID-19 confirmed by polymerase chain reaction (PCR) from nasal and pharyngeal samples during the study period of 29 February to 4 April 2020. Diagnostic confirmation was made in the hospital laboratories, as PCR is not available in primary healthcare centres. Signs and symptoms, the main available haematological and biochemical data and the results of imaging tests were recorded, as were comorbidities, the evolution, the hospitalization rate, intensive care unit (ICU) admission and the treatments received. The study population was divided into four age groups: 15–30 years, 31–49 years, 50–64 years and ≥65 years. Other variables recorded were the type of follow-up, the need for temporary work disability, and the source of possible contacts. The time to first medical visit was defined as the difference (in days) between symptom onset and medical visit by a family physician. The factors that determined a poor prognosis (hospitalization, ICU admission, death) were collected. The data were obtained from the electronic medical record. Missing data were collected by telephone interviews with patients when possible. Patients from nursing homes were excluded, as the rate of infections and mortality has been shown to be much higher than in the non-institutionalized population. The study was approved by the Ethics Committee of the Hospital Clinic of Barcelona (registration number HCB/2020/0525). The study was conducted according to the Helsinki Declaration and Spanish legislation on biomedical studies, data protection and respect for human rights.

### Statistical analysis

Categorical variables are presented as absolute frequencies and percentages (%) and continuous variables as means and standard deviations (SD). Predictors of death, ICU admission and hospitalization were determined using the student’s t test for continuous variables and the chi-square test for categorical variables. Logistic multivariate regression models adjusted for age and sex were constructed to analyse independent predictive factors associated with death, ICU admission and hospitalization. Odds ratios (OR) and their 95% confidence intervals (95%CI) obtained in the adjusted regression analysis were calculated. Forest plots were used to represent OR and 95%CI. Values of p<0.05 were considered statistically significant. The statistical analysis was performed using the R version 3.6.1. for Windows.

## Results

### Clinical characteristics and comorbidities

We included 322 patients (mean age 56.7 years, 50% female, 115 (35.7%) aged ≥ 65 years). The mean time from symptom onset to the medical visit was 3.9 (SD 4.6) days. Clinical characteristics are shown in Table 1.

**Table 1:** Clinical and exploratory factors predicting hospitalization and ICU admission/death.

Variables	Total	Death or ICU admission				Hospitalization			
	(n = 322)	No (n = 266)	Yes (n = 56)	*P*	Adjusted OR [95% CI]	No (n = 164)	Yes (n = 158)	*P*	Adjusted OR [95% CI]
**Age—years**	56.7 ± 17.8	54.3 ± 17.4	68.2 ± 14.9	<0.001	**1.05 [1.03–1.07]**	48.6 ± 16.0	65.1 ± 15.6	<0.001	**1.06 [1.04–1.08]**
Distribution—no. (%)				<0.001				<0.001	
15–30 years	32 (9.9)	30 (11.3)	2 (3.6)			30 (18.3)	2 (1.3)		
31–49 years	92 (28.6)	88 (33.1)	4 (7.1)			61 (37.2)	31 (19.6)		
50–64 years	83 (25.8)	71 (26.7)	12 (21.4)			46 (28.0)	37 (23.4)		
≥65 years	115 (35.7)	77 (28.9)	38 (67.9)			27 (16.5)	88 (55.7)		
**Male—no. (%)**	161 (50.0)	121 (45.5)	40 (71.4)	0.001	**2.94 [1.55–5.82]**	67 (40.9)	94 (59.5)	0.001	**2.08 [1.26–3.46]**
**Occupation—no. (%)**				<0.001				<0.001	
Other type of exposure	189 (58.7)	133 (50.0)	56 (100.0)		N/A	48 (29.3)	141 (89.2)		N/A
Health professional	123 (38.2)	123 (46.2)	0 (0)		N/A	106 (64.6)	17 (10.8)		N/A
Other health workers	10 (3.1)	10 (3.8)	0 (0)		N/A	10 (6.1)	0 (0)		N/A
**Smoking (ex-smoker/smoker)—no./total no. (%)**	81/260 (31.2)	60/212 (28.3)	21/48 (43.8)	0.057	1.45 [0.71–2.92]	31/119 (26.1)	50/141 (35.5)	0.109	1.13 [0.62–2.08]
**Temperature at admission— °C†**	37.6 ± 0.8	37.6 ± 0.8	37.8 ± 0.7	0.030	0.99 [0.95–1.04]	37.3 ± 0.8	37.9 ± 0.7	<0.001	**1.03 [1.00–1.08]**
Patients with fever (≥37.5°C)—no./total no. (%)	194/304 (63.8)	153/251 (61.0)	41/53 (77.4)	0.027	1.58 [0.77–3.43]	73/152 (48.0)	121/152 (79.6)	<0.001	**3.33 [1.89–5.96]**
**Time from symptom onset to medical visit—days‡**	3.9 ± 4.6	3.9 ± 4.8	3.9 ± 3.7	0.954	0.97 [0.90–1.04]	3.5 ± 4.9	4.3 ± 4.3	0.166	1.00 [0.95–1.06]
**Symptoms—no. (%)¶**									
Cough	238 (73.9)	198 (74.4)	40 (71.4)	0.620	0.76 [0.38–1.56]	114 (69.5)	124 (78.5)	0.076	1.47 [0.82–2.66]
General malaise	140 (43.5)	114 (42.9)	26 (46.4)	0.658	0.93 [0.50–1.74]	65 (39.6)	75 (47.5)	0.178	1.08 [0.65–1.79]
Fatigue	99 (30.7)	82 (30.8)	17 (30.4)	1.000	0.86 [0.43–1.66]	41 (25.0)	58 (36.7)	0.029	1.66 [0.96–2.89]
Myalgia or arthralgia	97 (30.1)	90 (33.8)	7 (12.5)	0.001	**0.31 [0.12–0.70]**	53 (32.3)	44 (27.8)	0.397	1.09 [0.63–1.90]
Dyspnoea	82 (25.5)	60 (22.6)	22 (39.3)	0.012	1.84 [0.95–3.52]	24 (14.6)	58 (36.7)	<0.001	**2.92 [1.62–5.42]**
Diarrhoea	74 (23.0)	64 (24.1)	10 (17.9)	0.384	0.59 [0.26–1.24]	29 (17.7)	45 (28.5)	0.024	1.77 [0.98–3.26]
Headache	67 (20.8)	62 (23.3)	5 (8.9)	0.018	0.55 [0.18–1.40]	37 (22.6)	30 (19.0)	0.493	1.64 [0.87–3.14]
Anosmia	56 (17.4)	53 (19.9)	3 (5.4)	0.006	0.51 [0.11–1.59]	45 (27.4)	11 (7.0)	<0.001	**0.42 [0.19–0.90]**
Dysgeusia	48 (14.9)	45 (16.9)	3 (5.4)	0.024	0.44 [0.10–1.36]	35 (21.3)	13 (8.2)	0.001	0.54 [0.24–1.14]
Sore throat	38 (11.8)	33 (12.4)	5 (8.9)	0.648	0.93 [0.29–2.52]	25 (15.2)	13 (8.2)	0.058	0.65 [0.29–1.44]
Blocked nose	38 (11.8)	34 (12.8)	4 (7.1)	0.360	0.74 [0.20–2.23]	24 (14.6)	14 (8.9)	0.122	0.90 [0.39–2.03]
Nausea or vomiting	38 (11.8)	30 (11.3)	8 (14.3)	0.500	1.44 [0.56–3.46]	15 (9.1)	23 (14.6)	0.167	1.54 [0.72–3.37]
Sputum production	29 (9.0)	21 (7.9)	8 (14.3)	0.130	2.00 [0.73–5.14]	13 (7.9)	16 (10.1)	0.561	1.22 [0.51–2.97]
Chills	21 (6.5)	17 (6.4)	4 (7.1)	0.770	0.72 [0.19–2.29]	4 (2.4)	17 (10.8)	0.003	**5.66 [1.68–23.49]**
Asthenia	12 (3.7)	10 (3.8)	2 (3.6)	1.000	0.72 [0.10–3.34]	3 (1.8)	9 (5.7)	0.081	3.29 [0.78–17.86]
Chest pain	9 (2.8)	7 (2.6)	2 (3.6)	0.658	2.88 [0.39–14.41]	7 (4.3)	2 (1.3)	0.174	0.39 [0.05–1.85]
**Alterations in physical examination—no./total no. (%)§**									
Auscultatory alterations	154/223 (69.1)	112/171 (65.5)	42/52 (80.8)	0.041	1.67 [0.77–3.86]	42/75 (56.0)	112/148 (75.7)	0.004	1.77 [0.93–3.34]
Tachypnoea	64/223 (28.7)	43/171 (25.1)	21/52 (40.4)	0.037	1.41 [0.70–2.81]	15/75 (20.0)	49/148 (33.1)	0.043	1.31 [0.65–2.72]
Tachycardia	29/223 (13.0)	23/171 (13.5)	6/52 (11.5)	0.818	0.86 [0.29–2.25]	7/75 (9.3)	22/148 (14.9)	0.296	2.07 [0.83–5.81]
Pharyngitis	22/223 (9.9)	22/171 (12.9)	0/52 (0)	0.003	N/A	9/75 (12.0)	13/148 (8.8)	0.480	0.98 [0.38–2.64]
**Oxygen saturation ≤92%—no./total no. (%)**	42/205 (20.5)	24/154 (15.6)	18/51 (35.3)	0.005	2.09 [0.96–4.52]	8/66 (12.1)	34/139 (24.5)	0.043	1.70 [0.73–4.33]

In bold, statistically significant independent predictive factors associated with hospitalization, death or ICU admission (logistic multivariate regression adjusted for age and sex).

† Temperature distribution was: <37.5°C (36.2%), 37.5–38.0°C (18.4%), 38.1–39.0°C (38.8%) and >39.0°C (6.6%).

‡ In 10 (3.1%) patients’ data on period between symptom onset and medical visit were lacking.

¶ Symptoms with a frequency of < 5 patients were: disorientation (n = 4), conjunctivitis (n = 3), haemoptysis (n = 2) and cutaneous lesions (n = 2).

§ 223 (69.3%) patients had a physical examination. The alterations with a frequency of < 5 patients were: cutaneous lesions (n = 2) and tonsillopharyngitis (n = 1).

Ref: reference, N/A: not applicable.

Notably, 123 (38.2) were health workers (doctors, nurses, auxiliaries). The most frequent clinical symptoms were cough (73.9%), fever (63.8%), general malaise (43.5%), fatigue (30.7%), myalgia or arthralgia (30.1%), dyspnoea (25.5%), diarrhoea (23%), headache (20.8%), anosmia (17.4%) and dysgeusia (14.9%). Physical examination in 223 (69.3%) patients showed 69.1% had auscultatory alterations, 28.7% tachypnoea and 20.5% an oxygen saturation of ≤ 92%.

**Fig 1:**
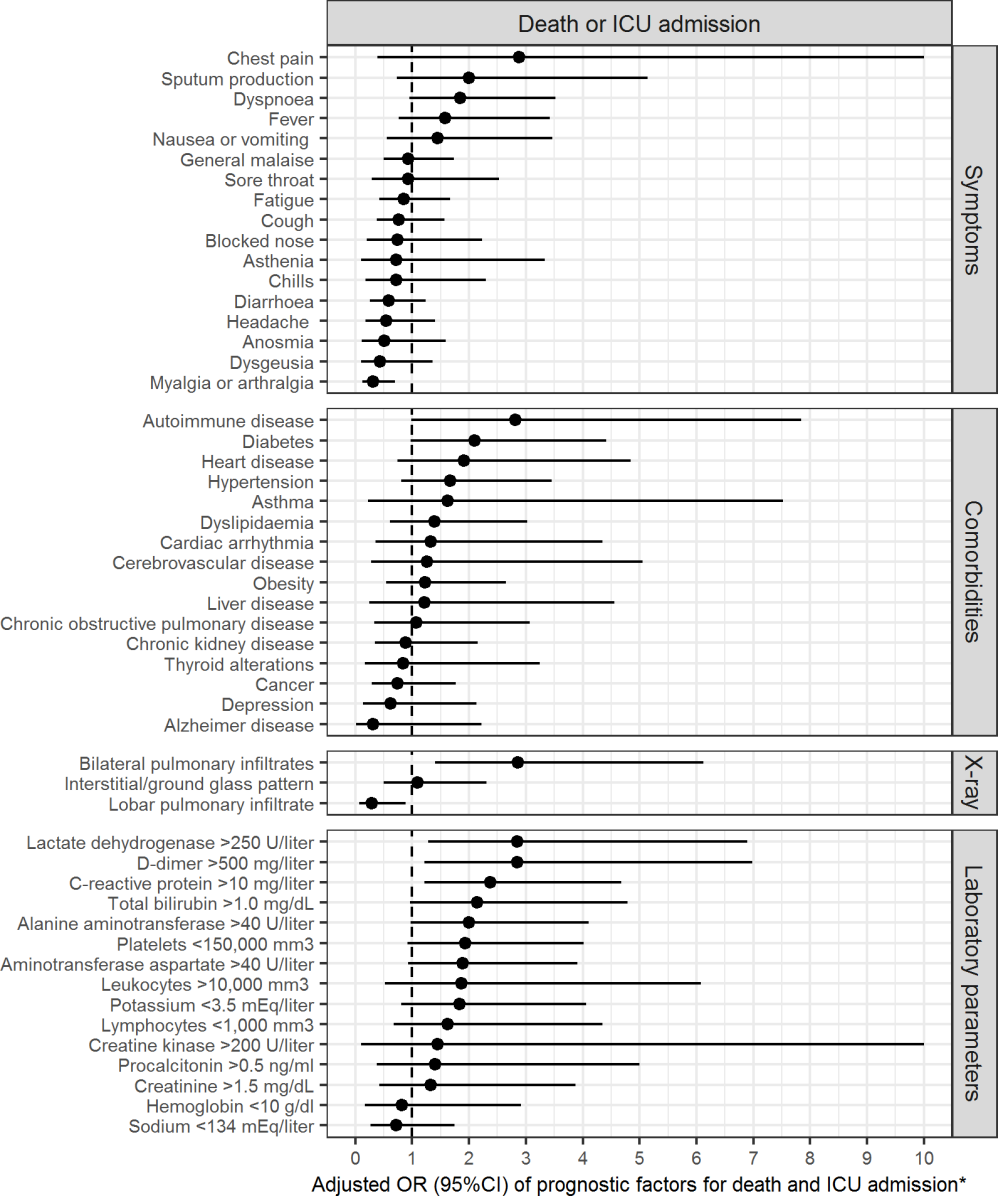
Prognostic factors for death and ICU admission. *The upper limits of the confidence intervals were restricted to 10 in order not to mask the significant effects of other variables with smaller ranges.

**Fig 2:**
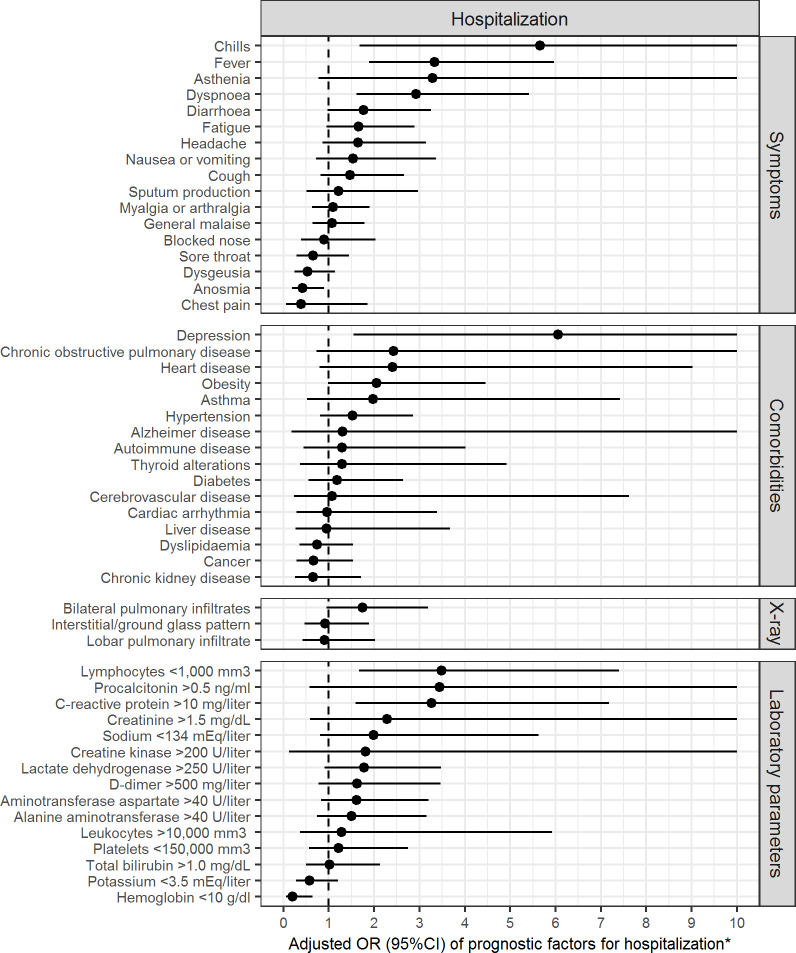
Prognostic factors for hospitalization. *The upper limits of the confidence intervals were restricted to 10 in order not to mask the significant effects of other variables with smaller ranges.

ICU admission and death were associated with a greater mean age (68.2 years vs 54.3 years, p < 0.001), male sex (71.4% vs 45.5%, p = 0.001), dyspnoea (39.3% vs 22.6%, p = 0.012), fever (77.4% vs 61.0%, p = 0.027), auscultatory alterations (80.8% vs 65.5%, p = 0.041) and low oxygen saturation (35.3% vs 15.6%, p = 0.005) (Table 1). Myalgia or arthralgia (12.5% vs 33.8%, p = 0.001), headache (8.9% vs 23.3%, p = 0.018), dysgeusia (5.4% vs 16.9%, p = 0.024) and anosmia (5.4% vs 19.9%, p = 0.006) were less frequent in patients admitted to the ICU or who died than the remaining patients. Age (OR = 1.05; 95%CI = 1.03 to 1.07) and male sex (OR = 2.94; 95%CI = 1.55 to 5.82) were independent predictors of ICU admission and death. Myalgia or arthralgia (OR = 0.31; 95%CI = 0.12 to 0.70) was the only significant protective factor against ICU admission and death after adjusting for age and sex (Fig 1). The best clinical predictors of hospitalization were chills (OR = 5.66; 95%CI = 1.68 to 23.49), fever (OR = 3.33; 95%CI = 1.89 to 5.96) and dyspnoea (OR = 2.92; 95%CI = 1.62 to 5.42). Anosmia (OR = 0.42; 95%CI = 0.19 to 0.90) was the only significant protective factor for hospitalization after adjusting for age and sex (Table 1 and Fig 2).

Comorbidities were presented by 212 (65.8%) patients: the most common were hypertension in 109 (33.9%), diabetes mellitus in 46 (14.3%), and obesity in 46 (14.3%) (Table 2). Heart disease (19.6% vs 5.3%, p = 0.001), autoimmune disease (16.1% vs 4.5%, p = 0.004), diabetes (32.1% vs 10.5%, p < 0.001), hypertension (60.7% vs 28.2%, p < 0.001) and chronic kidney disease (17.9% vs 7.9%, p = 0.042) were the comorbidities significantly associated with ICU admission and death (Table 2). Autoimmune disease was the only significant predictive comorbidity for ICU admission and death after adjusting for age and sex (OR = 2.82; 95%CI = 1.00 to 7.84) (Fig 1). Depression was the best predictor of hospitalization among all comorbidities (OR = 6.06; 95%CI = 1.54 to 40.42) (Fig 2). Having ≥ 1 comorbidity was associated with ICU admission and death (OR = 3.43; 95%CI = 1.30 to 10.83) and hospitalization (OR = 2.05; 95%CI = 1.13 to 3.75) independently of age and sex.

**Table 2:** Comorbidities associated with hospitalization and ICU admission/death.

Variables	Total	Death or ICU admission				Hospitalization			
	(n = 322)	No (n = 266)	Yes (n = 56)	*P*	Adjusted OR [95% CI]	No (n = 164)	Yes (n = 158)	*P*	Adjusted OR [95% CI]
**Comorbidities—no. (%)†**									
Any comorbidity	212 (65.8)	161 (60.5)	51 (91.1)	<0.001	**3.43 [1.30–10.83]**	82 (50.0)	130 (82.3)	<0.001	**2.05 [1.13–3.75]**
Hypertension	109 (33.9)	75 (28.2)	34 (60.7)	<0.001	1.67 [0.81–3.46]	29 (17.7)	80 (50.6)	<0.001	1.52 [0.81–2.86]
Diabetes	46 (14.3)	28 (10.5)	18 (32.1)	<0.001	2.10 [0.98–4.41]	13 (7.9)	33 (20.9)	0.001	1.19 [0.55–2.64]
Obesity	46 (14.3)	34 (12.8)	12 (21.4)	0.097	1.23 [0.54–2.65]	13 (7.9)	33 (20.9)	0.001	2.05 [0.99–4.46]
Dyslipidaemia	44 (13.7)	32 (12.0)	12 (21.4)	0.084	1.39 [0.61–3.03]	18 (11.0)	26 (16.5)	0.194	0.74 [0.36–1.54]
Cancer	37 (11.5)	28 (10.5)	9 (16.1)	0.250	0.75 [0.29–1.77]	13 (7.9)	24 (15.2)	0.054	0.66 [0.29–1.54]
Chronic kidney disease	31 (9.6)	21 (7.9)	10 (17.9)	0.042	0.89 [0.34–2.16]	9 (5.5)	22 (13.9)	0.013	0.65 [0.26–1.71]
Heart disease	25 (7.8)	14 (5.3)	11 (19.6)	0.001	1.92 [0.74–4.84]	4 (2.4)	21 (13.3)	<0.001	2.40 [0.80–9.02]
Autoimmune disease	21 (6.5)	12 (4.5)	9 (16.1)	0.004	**2.82 [1.00–7.84]**	7 (4.3)	14 (8.9)	0.116	1.30 [0.45–4.01]
Chronic obstructive pulmonary disease	19 (5.9)	13 (4.9)	6 (10.7)	0.114	1.07 [0.34–3.07]	3 (1.8)	16 (10.1)	0.002	2.43 [0.73–11.09]
Depression	18 (5.6)	15 (5.6)	3 (5.4)	1.000	0.62 [0.13–2.13]	2 (1.2)	16 (10.1)	<0.001	**6.06 [1.54–40.42]**
Cardiac arrhythmia	16 (5.0)	11 (4.1)	5 (8.9)	0.168	1.32 [0.36–4.34]	6 (3.7)	10 (6.3)	0.312	0.96 [0.29–3.39]
Thyroid alterations	14 (4.3)	11 (4.1)	3 (5.4)	0.717	0.84 [0.17–3.25]	5 (3.0)	9 (5.7)	0.283	1.29 [0.37–4.92]
Asthma	13 (4.0)	11 (4.1)	2 (3.6)	1.000	1.62 [0.22–7.52]	7 (4.3)	6 (3.8)	1.000	1.98 [0.52–7.43]
Liver disease	12 (3.7)	9 (3.4)	3 (5.4)	0.445	1.21 [0.25–4.55]	5 (3.0)	7 (4.4)	0.568	0.95 [0.27–3.68]
Cerebrovascular disease	10 (3.1)	6 (2.3)	4 (7.1)	0.076	1.26 [0.28–5.06]	2 (1.2)	8 (5.1)	0.057	1.08 [0.24–7.63]
Alzheimer disease	6 (1.9)	5 (1.9)	1 (1.8)	1.000	0.31 [0.02–2.22]	1 (0.6)	5 (3.2)	0.115	1.31 [0.18–26.68]

In bold, statistically significant independent predictive factors associated with hospitalization, death, or ICU admission (logistic multivariate regression adjusted for age and sex).

† Comorbidities with a frequency of < 5 patients were: bronchiectasis (n = 4), fibromyalgia (n = 4), anaemia (n = 3), arthritis (n = 3), HIV (n = 2), syphilis (n = 1) and tuberculosis (n = 1).

### Imaging and laboratory tests

Chest X-ray was necessary in 227 patients (70.5%) and showed lobar pulmonary infiltrates in 35 (15.4%), bilateral pulmonary infiltrates in 129 (56.8%) and an interstitial pattern in 48 (21.1%) (Table 3). Chest CT was required in 28 patients and pulmonary ultrasound in 10 (3.1%). Biologically, 171 (81.4%) of 210 patients had lymphopenia (< 1,000 mm3). Likewise, 60.8% had a lactate dehydrogenase (LDH) > 250 U/ml and liver test alterations were common: elevated AST/GOT in 41.4% and ALT/GPT in 32.4%. In 86 (52.1%) of 165 cases D-dimer was elevated (> 500mg/L). The most important factors for ICU admission and death were bilateral pulmonary infiltrates (OR = 2.86; 95%CI = 1.41 to 6.13), elevated lactate-dehydrogenase (OR = 2.85; 95%CI = 1.28 to 6.90), elevated D-dimer (OR = 2.85; 95%CI = 1.22 to 6.98) and elevated C-reactive protein (OR = 2.38; 95%CI = 1.22 to 4.68) (Fig 1). Significant predictive factors associated with hospitalization, after adjusting for age and sex, were lymphopenia (OR = 3.48; 95%CI = 1.67 to 7.40) and elevated C-reactive protein (OR = 3.27; 95%CI = 1.59 to 7.18) (Fig 2).

**Table 3:** Analytical and radiological predictors of hospitalization and ICU admission/death.

Variables	Total	Death or ICU admission (n = 56)				Hospitalization (n = 158)			
	(n = 322)	No (n = 266)	Yes (n = 56)	*P*	Adjusted OR [95% CI]	No (n = 164)	Yes (n = 158)	*P*	Adjusted OR [95% CI]
**Alterations in chest X-ray—no./total no. (%)†**									
Bilateral pulmonary infiltrates	129/227 (56.8)	86/172 (50.0)	43/55 (78.2)	<0.001	**2.86 [1.41–6.13]**	31/72 (43.1)	98/155 (63.2)	0.006	1.74 [0.95–3.19]
Interstitial/ground glass pattern	48/227 (21.1)	36/172 (20.9)	12/55 (21.8)	0.852	1.09 [0.50–2.31]	16/72 (22.2)	32/155 (20.6)	0.862	0.92 [0.46–1.89]
Lobar pulmonary infiltrate	35/227 (15.4)	32/172 (18.6)	3/55 (5.5)	0.018	**0.29 [0.07–0.88]**	13/72 (18.1)	22/155 (14.2)	0.438	0.91 [0.42–2.02]
**Alterations in chest CAT scan—no./total no. (%)‡**									
Bilateral pulmonary infiltrates	14/28 (50.0)	6/17 (35.3)	8/11 (72.7)	0.120	4.90 [0.84–38.73]	3/5 (60.0)	11/23 (47.8)	1.000	0.97 [0.10–8.82]
Interstitial/ground glass pattern	13/28 (46.4)	10/17 (58.8)	3/11 (27.3)	0.137	**0.01 [0.00–0.21]**	2/5 (40.0)	11/23 (47.8)	1.000	3.35 [0.28–64.12]
**Laboratory parameters—no./total no. (%)**									
Leukocytes >10,000 mm3	13/211 (6.2)	8/160 (5.0)	5/51 (9.8)	0.312	1.87 [0.53–6.08]	3/65 (4.6)	10/146 (6.8)	0.758	1.28 [0.37–5.92]
Lymphocytes <1,000 mm3	171/210 (81.4)	126/158 (79.7)	45/52 (86.5)	0.311	1.62 [0.68–4.35]	44/65 (67.7)	127/145 (87.6)	0.001	**3.48 [1.67–7.40]**
Platelets <150,000 mm3	46/208 (22.1)	28/156 (17.9)	18/52 (34.6)	0.020	1.94 [0.92–4.02]	11/63 (17.5)	35/145 (24.1)	0.364	1.22 [0.57–2.75]
Haemoglobin <10 g/dl	13/210 (6.2)	10/158 (6.3)	3/52 (5.8)	1.000	0.82 [0.17–2.92]	8/64 (12.5)	5/146 (3.4)	0.024	**0.20 [0.06–0.65]**
C-reactive protein >10 mg/litre	78/210 (37.1)	49/159 (30.8)	29/51 (56.9)	0.001	**2.38 [1.22–4.68]**	11/63 (17.5)	67/147 (45.6)	<0.001	**3.27 [1.59–7.18]**
Procalcitonin >0.5 ng/ml	13/111 (11.7)	7/79 (8.9)	6/32 (18.8)	0.191	1.40 [0.38–5.00]	1/28 (3.6)	12/83 (14.5)	0.178	3.44 [0.57–66.4]
Lactate dehydrogenase >250 U/litre	115/189 (60.8)	81/146 (55.5)	34/43 (79.1)	0.007	**2.85 [1.28–6.90]**	26/54 (48.1)	89/135 (65.9)	0.032	1.78 [0.91–3.47]
Aminotransferase aspartate >40 U/litre	79/191 (41.4)	55/147 (37.4)	24/44 (54.5)	0.055	1.89 [0.92–3.91]	18/56 (32.1)	61/135 (45.2)	0.108	1.61 [0.83–3.20]
Alanine aminotransferase >40 U/litre	61/188 (32.4)	41/143 (28.7)	20/45 (44.4)	0.067	2.00 [0.97–4.11]	14/53 (26.4)	47/135 (34.8)	0.302	1.50 [0.74–3.16]
Total bilirubin >1.0 mg/dL	58/170 (34.1)	41/133 (30.8)	17/37 (45.9)	0.116	2.14 [0.97–4.79]	17/48 (35.4)	41/122 (33.6)	0.858	1.02 [0.49–2.14]
Creatine kinase >200 U/litre	4/23 (17.4)	2/15 (13.3)	2/8 (25.0)	0.589	1.45 [0.10–20.20]	1/7 (14.3)	3/16 (18.8)	1.000	1.81 [0.12–55.29]
Creatinine >1.5mg/dL	17/210 (8.1)	10/158 (6.3)	7/52 (13.5)	0.139	1.32 [0.43–3.87]	2/63 (3.2)	15/147 (10.2)	0.103	2.28 [0.58–15.17]
D-dimer >500 mg/litre	86/165 (52.1)	60/128 (46.9)	26/37 (70.3)	0.015	**2.85 [1.22–6.98]**	18/44 (40.9)	68/121 (56.2)	0.112	1.63 [0.77–3.47]
Sodium <134 mEq/litre	33/211 (15.6)	26/160 (16.2)	7/51 (13.7)	0.826	0.72 [0.27–1.75]	6/64 (9.4)	27/147 (18.4)	0.148	1.98 [0.81–5.63]
Potassium <3.5 mEq/litre	41/202 (20.3)	28/156 (17.9)	13/46 (28.3)	0.145	1.83 [0.81–4.06]	16/61 (26.2)	25/141 (17.7)	0.185	0.57 [0.27–1.21]

In bold, statistically significant independent predictive factors associated with hospitalization, death or ICU admission (logistic multivariate regression adjusted for age and sex).

† 227 (70.5%) patients had a chest X-ray. The alterations with a frequency < 5 patients were: pneumothorax (n = 2) and pleural effusion (n = 1). Chest X-ray results were not available in 5 patients.

‡ 28 (8.7%) patients had a chest CAT scan. Alterations with a frequency of < 5 patients were: pulmonary thromboembolism (n = 4), emphysema (n = 2), lobar pulmonary infiltrates (n = 2), pneumonia (n = 2), atelectasis (n = 2) and pleural effusion (n = 1). CAT scan results were not available in five patients.

### Treatment, complications and evolution

Treatment included hydroxychloroquine in 162 (50.3%) patients, azithromycin in 149 (46.3%), lopinavir/ritonavir in 132 (40.7%), glucocorticoids in 34 (10.6%) and tocilizumab in 27 (8.4%), among others (Table 4), and 49.1% of patients required hospitalization. Phone follow up was registered in 277 (86.0%) patients, 57 (17.7%) patients were monitored at home. 161 (77.8%) of the 207 patients of working age sought work disability due to COVID-19. The ICU admission rate was 13.0%. The evolution included pneumonia in 177 (55%) patients, adult respiratory distress syndrome in 37 (11.5%), severe renal failure in 8 (2.5%), pulmonary thromboembolism in 4 (1.2%) and sepsis in 3 (0.9%) patients. Occupational contact with persons with confirmed or suspected COVID-19 infection was reported by 71 (22.0%) patients, while 51 (15.8%) reported that contact occurred in the family setting. Occupational contact was a protective factor against hospitalization (OR = 0.41; 95%CI = 0.20 to 0.80), ICU admission and death (OR = 0.12; 95%CI = 0.01 to 0.59) after adjusting for age and sex. The mortality rate to date was 5.6%.

**Table 4:** Predictors of the evolution, complications and treatment in patients hospitalized or with ICU admission/death.

Variables	Total	Death or ICU admission (n = 56)				Hospitalization (n = 158)			
	(n = 322)	No (n = 266)	Yes (n = 56)	*P*	Adjusted OR [95% CI]	No (n = 164)	Yes (n = 158)	*P*	Adjusted OR [95% CI]
**Complications—no. (%)†**									
Any complication	195 (60.6)	139 (52.3)	56 (100.0)	<0.001	N/A	47 (28.7)	148 (93.7)	<0.001	**22.64 [10.72–52.30]**
Pneumonia	177 (55.0)	128 (48.1)	49 (87.5)	<0.001	**3.79 [1.64–9.91]**	44 (26.8)	133 (84.2)	<0.001	**8.37 [4.69–15.29]**
Adult respiratory distress syndrome	37 (11.5)	6 (2.3)	31 (55.4)	<0.001	**43.51 [16.51–134.77]**	10 (6.1)	27 (17.1)	0.003	1.39 [0.60–3.41]
Renal failure	8 (2.5)	4 (1.5)	4 (7.1)	0.033	2.58 [0.56–11.89]	1 (0.6)	7 (4.4)	0.034	3.25 [0.52–63.61]
Pulmonary thromboembolism	4 (1.2)	3 (1.1)	1 (1.8)	0.536	0.99 [0.05–8.42]	0 (0)	4 (2.5)	0.057	N/A
**Treatments—no. (%)‡**									
Hydroxychloroquine	162 (50.3)	125 (47.0)	37 (66.1)	0.012	1.36 [0.71–2.64]	45 (27.4)	117 (74.1)	<0.001	**5.74 [3.36–9.98]**
Azithromycin	149 (46.3)	120 (45.1)	29 (51.8)	0.380	0.74 [0.39–1.41]	42 (25.6)	107 (67.7)	<0.001	**4.34 [2.56–7.46]**
Lopinavir/Ritonavir	131 (40.7)	100 (37.6)	31 (55.4)	0.017	1.34 [0.71–2.51]	30 (18.3)	101 (63.9)	<0.001	**6.05 [3.49–10.72]**
Oxygen therapy	86 (26.7)	48 (18.0)	38 (67.9)	<0.001	**6.35 [3.24–12.79]**	18 (11.0)	68 (43.0)	<0.001	**3.50 [1.87–6.79]**
Intravenous antibiotics	77 (23.9)	48 (18.0)	29 (51.8)	<0.001	**3.01 [1.57–5.78]**	18 (11.0)	59 (37.3)	<0.001	**2.64 [1.41–5.10]**
Glucocorticoids	34 (10.6)	16 (6.0)	18 (32.1)	<0.001	**4.51 [2.04–10.06]**	5 (3.0)	29 (18.4)	<0.001	**3.39 [1.3–10.64]**
Tocilizumab	27 (8.4)	17 (6.4)	10 (17.9)	0.013	2.15 [0.86–5.14]	7 (4.3)	20 (12.7)	0.008	1.72 [0.69–4.72]
Cephalosporins	22 (6.8)	18 (6.8)	4 (7.1)	1.000	0.60 [0.16–1.75]	1 (0.6)	21 (13.3)	<0.001	**13.57 [2.68–247.87]**
Low molecular weight heparin	19 (5.9)	12 (4.5)	7 (12.5)	0.030	2.30 [0.79–6.32]	1 (0.6)	18 (11.4)	<0.001	**15.81 [2.93–296.28]**
Remdesivir	6 (1.9)	4 (1.5)	2 (3.6)	0.280	2.64 [0.32–15.92]	2 (1.2)	4 (2.5)	0.441	2.03 [0.33–16.33]
**Covid-19 infection—no. (%)**									
Any cohabitant	51 (15.8)	39 (14.7)	12 (21.4)	0.227	1.21 [0.53–2.61]	25 (15.2)	26 (16.5)	0.879	0.70 [0.34–1.40]
Any work colleague	71 (22.0)	70 (26.3)	1 (1.8)	<0.001	**0.12 [0.01–0.59]**	57 (34.8)	14 (8.9)	<0.001	**0.41 [0.20–0.80]**
Any contact person in other settings	11 (3.4)	6 (2.3)	5 (8.9)	0.026	2.43 [0.62–9.29]	2 (1.2)	9 (5.7)	0.032	2.47 [0.55–17.84]

In bold, statistically significant independent predictive factors associated with hospitalization, death, or ICU admission (logistic multivariate regression adjusted for age and sex).

† Complications in < 5 patients were: sepsis (n = 3), multiorgan failure (n = 2), electrolyte alterations (n = 2), hematologic alterations (n = 2) and lung cancer (n = 1).

‡ Treatments with a frequency of < 10 patients, except remdesivir, were: amoxicillin (n = 6), interferon (n = 5), rituximab (n = 5), darunavir (n = 2) and entecavir (n = 1).

N/A: not applicable.

## Discussion

This study summarizes the clinical, biological and radiological characteristics, evolution and prognostic factors of patients with COVID-19 disease in primary and community healthcare. To date, we are aware of three published Spanish studies [10–12]. The first reported data from 48 patients on ICU admissions in a region where the pandemic was reported early [10]. The study by Borobia et al [11] describes the first 2226 adult patients with COVID-19 consecutively admitted to a University Hospital in Madrid. The third focuses on the differences by age-dependent categories in the clinical profile, presentation, management, and short-term outcomes [12]. Although there have been two systematic reviews and meta-analysis that analyse the clinical characteristics of COVID-19, they are limited to Chinese cohorts or case series [13, 14] and a large USA cohort [15] that did not analyse clinical predictors of a poor prognosis.

Clinically, the same main symptoms of cough and fever are reported in all series. However, in Barcelona city, we have observed diarrhoea, anosmia and dysgeusia, which is hardly reported in the Chinese series [7] which, unlike ours comes principally from hospitals: diarrhoea occurred in 23.8% of cases, very similar to the 23% in New York [16] and clearly higher than the 3.8% reported in China. Nearly 20% of patients had anosmia and dysgeusia, similar to the results obtained in French patients [17]. In contrast, expectoration was found in only 9%, compared with 33.7% in the Chinese series.

Chinese patients had a mean age of 47 years, ten years lower than our series, and 35.7% of our patients were aged ≥ 65 years, compared with 15%, 29% and 31% in China, Germany and the USA respectively, but > 40% in Italy [7, 18–20]. Older age and male sex predisposed to a higher mortality rate in our and all large series [7, 15]. In our patients, comorbidities were three times higher than in the Chinese cohort [7] and were similar to the findings of the New York study [15]. Any comorbidity was a risk factor for hospitalization, ICU admission and death. Depression was an independent risk factor for hospitalization, which has not been observed in other cohorts studied. Depression was often accompanied by a vulnerable social situation, which may have justified hospitalization. Likewise, autoimmune diseases were independent risk factor for ICU admission and death. Various hypotheses have been postulated on possible autoimmune alterations in the pathogenic evolution of the disease. With respect to treatment, no drug has proved effective against Covid-19 until now. Moreover, many treatments were unavailable in the outpatient setting. Currently, we are only certain that treatment with tocilizumab showed better survival rates in retrospective cohorts [21], although its efficacy has not been tested in randomized clinical trials. Therefore, the results on the outcomes associated with treatment should be interpreted with caution.

The same comorbidities were identified, with hypertension and diabetes being the two most common, while in the USA and Italy, obesity seems to be higher. Our results show that obesity was close to being an independent risk factor for hospitalization (OR = 2.05; 95%CI = 0.99 to 4.46).

Strikingly, 38.2% of our patients were healthcare workers, compared with 3.5% in Wuhan and 5.2% in Germany [7, 18]. Although these studies recognized an important degree of underreporting of cases in health workers, the difference remains important. There are at least two possible explanations: first, the lack of personal protective equipment in the initial phase of the epidemic, a constant revindication of health professionals, who felt undersupplied. Secondly, many cases were health professionals from primary healthcare or the reference hospital who reside in the same area where they work.

In all reported series, bilateral pneumonia was the most common radiological finding, was present in more than half the cases [22] and was a factor of a poor prognosis and mortality. In contrast, an interstitial radiological pattern did not confer an increased risk of mortality. The Wuhan study reported a CAT scan use of 88.7%, compared with 8.7% in Barcelona. In contrast, chest X-rays were carried out in 59.1% and 70.5%, respectively: the availability of diagnostic means was higher in China. A recent international consensus states that radiological assessment is not necessary in asymptomatic patients or those with mild disease but is required in patients with moderate or severe disease, regardless of whether a definite diagnosis of COVID-19 has been made [23]. In addition, simple chest X-rays are preferable in a resource-constrained environment with difficulties in accessing CAT scans [23]. The possible use of pulmonary ultrasound for the point-of-care diagnosis of COVID-19 pneumonia has not been sufficiently analysed but might be an efficient alternative due to its portability and reliability [24]. In fact, the regional Catalan government has recently acquired 90 ultrasound machines to enable family physicians to make doctors can make point-of-care (home or nursing home) diagnoses of pneumonia [25]. Biologically, lymphopenia and increased CRP, LDH and D-dimer were usually constant and similar in all series and were associated with an increased risk of mortality. A differential variable in our series is a greater number of alterations in liver tests, which was present in 30–40% of patients, data similar to the USA and Italian cohorts, but different from the Chinese cohort, where it was 22% [7]. We also found hypokalaemia in 20.5% of patients, a factor not reported in other studies.

We found a hospitalization rate of 48.7%, compared with 20–31% in the USA and 93.6% in China, and an ICU admission rate of 13%, which was similar to the Chinese (15%), USA (5–11.5%) and German (10%) results. While the protocols of action and admission are similar and depend on the level of clinical involvement, the therapeutic protocols differ between hospitals, cities, and countries. There remain many unknowns in the treatment of COVID-19. The only truth is that we do not have a vaccine, an etiological treatment or a treatment with sufficient scientific evidence to generalize its use. Currently, the systematic review of antiretroviral treatments has not offered conclusive results [26] and despite in vitro results for hydroxychloroquine, COVID-19 infections are currently intractable [27, 28].

The mortality rate in our study was 5.6%, compared with 10.2% in New York (21% in hospitalized patients), 1.4% in China, 3.1% in Germany and 6.8% in Italy. Different information and recording systems, the availability of diagnostic tests, and above all, the organization of national health systems may have contributed to the differences observed.

The study had some limitations due to the observational, retrospective design. However, it is sufficiently representative of the population with confirmed COVID-19 to permit better identification of the factors of a poor prognosis of the disease from a clinical perspective. We cannot rule out some heterogeneity in data codification due to observers’ interpretations of the medical records. However, this bias is minimal, as most clinical factors included are clearly defined in the electronic medical record. Another limitation of this study is the percentage of patients without laboratory parameters (more than 30%). Even though in real clinical practice these percentages may be expected, the results corresponding to laboratory parameters should be interpreted with caution.

Four months after the declaration of the pandemic, there is not a sufficiently reliable, available and generalizable diagnostic test that can analyse the seroprevalence of COVID-19, even in the most industrialized countries. Given this lack, determining the clinical, biological and radiological characteristics of probable cases of COVID-19 infection will be key to the initiation of early treatment and isolation, and for contact tracing, especially in primary healthcare.

## Supporting information

S1 Dataset (XLSX)
